# Preterm birth in evolutionary context: a predictive adaptive response?

**DOI:** 10.1098/rstb.2018.0121

**Published:** 2019-02-25

**Authors:** Thomas C. Williams, Amanda J. Drake

**Affiliations:** 1 MRC Human Genetics Unit, Institute of Genetics and Molecular Medicine, University of Edinburgh, Edinburgh EH4 2XU, UK; 2 British Heart Foundation Centre for Cardiovascular Science, University of Edinburgh, The Queen's Medical Research Institute, Edinburgh EH16 4TJ, UK

**Keywords:** preterm birth, early life effects, predictive adaptive response, evolution

## Abstract

Preterm birth is a significant public health problem worldwide, leading to substantial mortality in the newborn period, and a considerable burden of complications longer term, for affected infants and their carers. The fact that it is so common, and rates vary between different populations, raising the question of whether in some circumstances it might be an adaptive trait. In this review, we outline some of the evolutionary explanations put forward for preterm birth. We specifically address the hypothesis of the predictive adaptive response, setting it in the context of the Developmental Origins of Health and Disease, and explore the predictions that this hypothesis makes for the potential causes and consequences of preterm birth. We describe how preterm birth can be triggered by a range of adverse environmental factors, including nutrition, stress and relative socioeconomic status. Examining the literature for any associated longer-term phenotypic changes, we find no strong evidence for a marked temporal shift in the reproductive life-history trajectory, but more persuasive evidence for a re-programming of the cardiovascular and endocrine system, and a range of effects on neurodevelopment. Distinguishing between preterm birth as a predictive, rather than immediate adaptive response will depend on the demonstration of a positive effect of these alterations in developmental trajectories on reproductive fitness.

This article is part of the theme issue ‘Developing differences: early-life effects and evolutionary medicine'.

## 1. Introduction

Prematurity (delivery at a gestational age of less than 37 weeks) [1] affects 11% of live births worldwide. The sequelae of preterm birth constitute a significant public health problem, with preterm birth complications responsible for 35% of neonatal deaths (in the first 28 days of life) worldwide [2], and contributing to 50% of all deaths in this age group. In addition to death in the neonatal period, preterm birth is associated with a range of longer-term physical and neurodevelopmental consequences such as moderate/severe cognitive impairment, motor impairment and cerebral palsy [2]. It leads to an estimated annual 106 million disability-adjusted life years [3], and thus places a significant burden on parents, carers and health systems [4]. Why preterm birth should be so common (up to 18% of all live births in countries such as Malawi [5]), despite the fact that the consequences can be so catastrophic, remains unclear.

In this review, we will outline the hypotheses that have been advanced that place the phenomenon of human preterm birth in an evolutionary context. After discussing these, we will focus specifically on addressing the hypothesis that preterm birth may, in some circumstances, be a *predictive,* adaptive, response to adverse *in utero* conditions for the fetus. In order to do so, we start by situating preterm birth in the context of low birthweight (LBW) and the Developmental Origins of Health and Disease (DOHaD) [6]. Following this, we locate preterm birth in intra-species perspective, and examine the range of gestations likely to have been viable prior to the advent of modern medical interventions. We discuss whether some of the known triggers for preterm birth fit within an evolutionary framework, and the evidence that suggests that preterm birth may form part of a suite of predictive responses to an adverse *in utero* environment. We conclude by examining the limitations of the data, and ask whether prematurity should be best seen as an immediate, or predictive, adaptive response.

## 2. Could preterm birth represent an adaptive response to adverse conditions?

The null hypothesis for preterm birth having any adaptive significance is that it represents a pathophysiological response to triggers such as systemic or intra-uterine infections, or the endothelial dysfunction associated with diseases such as pre-eclampsia. From this perspective, preterm birth could be seen as a pathological process that has no evolutionary context or implications.

Another argument that has been put forward is that preterm birth has no adaptive advantage for the fetus, but that premature delivery, associated with a low chance of survival for the fetus, instead allows a mother to prioritize her own survival, and increase her chances of having a subsequent, more successful pregnancy [7].

A more nuanced alternative to this is that preterm delivery may provide an *immediate* adaptive advantage to either the fetus or the mother, but that the length of gestation is likely to represent the outcome of a trade-off, for both the fetus and mother, between the benefits and costs of continuing a pregnancy. It has long been recognized that for many sexually reproducing species, and in particular for non-human primates, at certain points during gestation and early life there is likely to be conflict between the interests of the mother and the fetus/infant, in relation to both the length and amount of parental investment [8]. This idea has been further developed by Haig [9], who argues that a fetus throughout its existence is likely to want to maximize parental investment, initially through the longest gestation possible, at least while *in utero* conditions are more favourable than those after delivery. However, throughout gestation a mother's reproductive decisions are more likely to be influenced by a trade-off between the chance of a fetus surviving birth and thus transmitting maternal alleles, and the fitness costs of continuing a gestation beyond an optimal duration (for the mother), particularly during the final weeks, which are associated with the deposition of subcutaneous fat in the fetus and substantial energetic investment from the mother [9].

Thus, faced with adverse intra-uterine circumstances, a fetus may choose to trigger delivery (as is seen with intra-uterine infection or with growth restriction [10]) prematurely; conversely, beyond a certain point in gestation for a mother in adverse circumstances, preterm birth may represent the chance to optimize the chance of survival for her offspring. Within this framework, ‘human developmental plasticity enables the alignment of offspring developmental trajectory with maternal phenotype’ [11, p. 332].

A final possibility, which we examine in detail in this paper, is that preterm birth may form one of a suite of predictive adaptive responses (PAR) to an adverse environment: the information provided by the maternal niche to the fetus about the *ex utero* environment leads to changes in developmental trajectories appropriate to this environment.

## 3. The PAR hypothesis

The PAR hypothesis refers to ‘a form of developmental plasticity in which cues received in early life influence the development of a phenotype that is normally adapted to the environmental conditions of later life’ [12, p. 2357]. A classic example has been described in the freshwater crustacean *Daphnia pulex*. If a predatory midge is present in the environment antenatally, the offspring develop a spiked helmet and long pointed tail, which provide protection against the midge, but are associated with reduced reproductive success if the predators disappear before reproductive age is attained. A PAR is also documented in voles (*Microtus pennsylvanius*), where pups born in the autumn have thicker coats than those born in spring; the cue to produce a thicker coat is provided by hormonal signals from the mother before birth, determined by day length [13].

A possible example of a PAR in humans is seen in cases of kwashiorkor and marasmus, both forms of malnutrition, where the development of each disease was associated in Jamaican children with different birthweights. Those with marasmus had a lower birthweight than those with kwashiorkor, but were also less likely to die during an acute episode of malnutrition, suggesting a possible *in utero*-induced PAR appropriate to the low-nutritional conditions that had induced their birthweight [14]; these individuals also showed persistence of differences in metabolic control into adulthood [15].

## 4. Predictions for preterm birth as part of a PAR

Placing LBW in evolutionary context, Gluckman and co-workers [16,17] have argued for phenotypic adaptations to adverse *in utero* conditions as an adaptive developmental response. They hypothesize that the capacity to alter the trajectory of development, and thus the mature phenotype, in response to environmental influences, may lead to increased chances of survival and reproduction. These adaptive responses may be immediate—that is, challenges to which an individual must respond immediately—or predictive, where fitness is enhanced by matching the phenotype better to the anticipated environment [18]. The conditions that a fetus is responding to are represented by the mother's phenotype, who in this view acts as an ‘integrating transducer of environmental information’ [18, p. 88].

**Figure 1.**
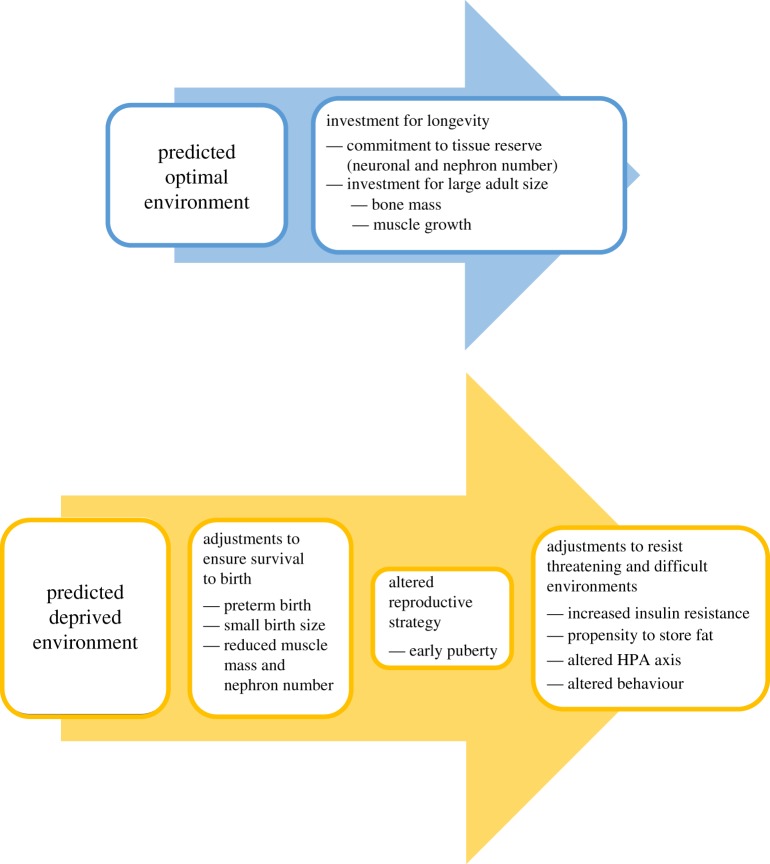
Preterm birth as part of a predictive adaptive response (adapted with the authors' permission from fig. 4 in [16]). (Online version in colour.)

In this theoretical framework, preterm birth forms part of a suite of potential developmental trajectories in response to a perceived deprived environment and a predicted uncertain life course [16]. Preterm birth, together with small birth size and reduced investment in tissues such as nephrons and muscle, forms part of the adjustments to ensure survival to birth. It is associated with an altered reproductive strategy, and is also related to adjustments to resist threatening and difficult environments: an altered hypothalamic–pituitary–adrenal (HPA) axis, altered behaviour, increased insulin resistance and a propensity to store fat (figure 1).

To examine whether preterm birth could be seen as a PAR, we first discuss initial findings showing that early life exposures correlate with later cardiometabolic outcomes, and the place of preterm birth within these cohorts. We then examine the data available from which to infer preterm birth rates in the pre-industrial context in which most of human evolution has taken place. Having outlined the evidence base available for the subsequent section, we move on to examine specific aspects of the PAR hypothesis in relation to preterm birth.

## 5. Preterm birth and low birth weight

It has been recognized since the first half of the twentieth century that early life exposures might have an influence on outcomes in later life [19,20]. However, it was Barker and colleagues who first explicitly linked birthweight to the risk of adverse outcomes in adulthood. They studied a cohort of 7991 men born in Hertfordshire, England [21] from 1911 to 1930, and found an inverse association between birthweight and risk of death from heart attack or stroke, with the effect most marked in those with a birthweight of less than 2.5 kg. Subsequent studies have confirmed these findings [22] in both high- and low-income settings. Over time these findings crystallized into the DOHaD hypothesis: the idea that the ‘risk of developing some chronic non-communicable diseases in adulthood is influenced not only by genetic and adult lifestyle factors but also by environmental factors acting in early life’ [6, p. 1733].

While in most contemporary settings gestational age is calculated using the date of the start of the last menstrual period (LMP), or in higher-income locations ultrasound measurements, birthweight was used throughout much of the twentieth century to identify infants born preterm. In 1919, prematurity was defined as a birthweight of less than 2500 g [23]. This somewhat arbitrary weight cut-off was formalized in a 1935 meeting of the American Association of Pediatrics [24], and in 1948 by the First World Health Assembly [25]. Thus, many of the initial studies looking in historical cohorts at the relationship between birthweight and later adverse outcomes did not distinguish between LBW owing to prematurity, and LBW in infants born at term (at a gestation of 37 weeks or more) owing to poor fetal growth. A 1928 study from the USA [26] (contemporaneous with the birth of members of the Hertfordshire cohort) states that of a group of infants with a birthweight of less than 2500 g, 72% were ‘premature’, suggesting that a significant proportion of the high-risk individuals in the Hertfordshire cohort were born preterm. More recently, a study examining the annual 18 million LBW (defined as less than 2500 g) [1] infants born worldwide estimated that 41% were preterm [27], again showing a significant overlap in the LBW group between preterm infants with a gestationally appropriate birthweight, and LBW term infants.

It is therefore likely that many of the cohorts used to analyse the long-term consequences of LBW comprise a heterogeneous population of preterm infants with and without growth restriction, and term infants with growth restriction (for definitions of these terms, see table 1).

**Table 1.** Definitions used in the review. All come from the World Health Organization [1,28,29].

term	definition
preterm birth	live birth at a gestation of less than 37 weeks, or up to and including 36 weeks and 6 days, starting from the date of the onset of the LMP
term birth	live birth at a gestation of between 37 and 42 weeks
moderately preterm birth	live birth at gestation of 32 to less than 37 weeks
very preterm birth	live birth at gestation of 28 to less than 32 weeks
extremely preterm birth	live birth at gestation < 28 weeks
low birthweight	birthweight < 2500 g
very low birthweight	birthweight < 1500 g
extremely low birthweight	birthweight < 1000 g
small for gestational age	birthweight below the 10th percentile of the recommended sex-specific birthweight for gestational age reference curves, often caused by fetal growth restriction
fetal growth restriction	a common pregnancy condition in which the fetus does not reach their biological growth potential, most often because of placental dysfunction; also referred to as intra-uterine growth retardation

 For example, in 18 cohorts included in a meta-analysis examining the long-term effects of birthweight on ischaemic heart disease [22], only five accounted for gestational age. Thus, rather than being a special case within the group, preterm birth is a significant contributor to LBW, and the findings for those born preterm have implications for the DOHaD hypothesis as a whole.

## 6. Defining preterm birth: do other species show preterm birth, and what are the limits of viability?

An important point is whether survival following preterm birth is likely to have an evolutionary context, as without modern medical support death rates for preterm infants are high. The first question that arises is whether preterm birth is unique to humans—that is, is the distribution of gestations at which live births occur wider than in other species? A cross-species comparative study using a cut-off of less than 92.5% of the mean gestation (equivalent to less than 37/40 weeks in humans) found evidence of a similar distribution of relative gestational ages in which live births occurred in a number of non-human primates [30]. Another study found that if preterm birth was defined as 2 s.d. below the mean (equivalent to gestation of less than 36 weeks in humans) [25], 16% of chimpanzees could be classified as having been born preterm [31]. The authors of the comparative study conclude that many mammals give birth before the ‘optimal period’, and thus modulation of birth timing may be a way in which individuals can adapt reproductive behaviour to environmental conditions; this would be in keeping with Haig's concept of trade-offs during gestation [9]. This has been explicitly tested in sheep, a commonly used animal model for human preterm birth, where severe malnutrition during pregnancy leads to a shortening of mean gestation (by 6%) [32], whereas moderate malnutrition leads to lengthening of gestation in some study subgroups [33].

The second key question is at what gestation infants are viable without any medical intervention. The importance of this intervention at extremely early gestations (extremely preterm, table 1) is starkly demonstrated by the difference in survival rates for gestations of 28–32 weeks in high-income countries (approx. 95%) compared with those in low-income countries (estimated at 38.6% for the first 7 days of life in a multi-centre study from 2010) [34]. In a historical setting, a 1902 paper, before the advent of modern intensive care, states that ‘everyone accepts the survival of an infant at seven months' (i.e. equivalent to 30 weeks) [35, p. 1197]. A 1955 study [36], before the widespread introduction of invasive mechanical ventilation, found survival through the neonatal period for gestations as low as 25 weeks, and survival rates through the neonatal period of roughly 50% for birth at 30 weeks. Similarly, while mortality is high, studies in low-income settings such as Malawi with minimal medical infrastructure for preterm infants show that neonatal survival rates for infants with a birthweight of between 2000 and 2500 g are 95%, with rates of approximately 50% for those born at a birthweight of 1000–1500 g (roughly equivalent to a gestational age of approx. 30 weeks) [37]. In both developed and developing settings, the majority (approx. 85%) of preterm births are classified as moderately preterm (32 to less than 37 weeks) [5], where in general survival is high and often no medical input is required [38]. A meta-analysis of data from low-income countries suggests that the risk of neonatal death in this group is only twice that of the baseline population neonatal mortality rate [4]. In summary, while mortality clearly increases with birth at earlier gestation, survival is possible even at very early gestations and likely at moderate prematurity [28].

## 7. Preterm birth as a response to adverse life conditions

Locating preterm birth as part of a predictive adaptive response implies that it could be triggered by an adverse maternal environment. The aetiology of preterm birth includes a heterogeneous range of causes, some specific to a modern medical context. Deliveries may be medically expedited for maternal diseases such as pre-eclampsia, or following evidence of maternal ascending infection (chorio-amnionitis); infection in itself may act as a trigger to preterm birth [39]. However, the most common group of preterm births occur owing to the spontaneous onset of labour in the absence of clear pathological precipitating factors. A number of associations with spontaneous preterm birth have been identified, including race [39], a personal or family history of preterm delivery and markers of maternal stress such as anxiety or depression, low maternal socioeconomic and educational status, and low maternal body mass index (BMI).

Recent studies examining the relationship between maternal BMI and gestational duration show an association between lower pre-pregnancy maternal weight and a higher risk of giving birth prematurely after spontaneous onset of labour [40,41]. High levels of maternal stress are also linked to an increased risk of LBW, shown by studies looking at birth outcomes after natural disasters [42]. Mothers experiencing high levels of psychological or social stress [43], or exposed to challenging conditions such as poor housing or poverty [44], are also at a higher risk of giving birth prematurely. Another marker of maternal wellbeing is socioeconomic status (SES)—it is recognized in humans and non-human primates that it is not necessarily absolute living conditions, but those relative to other individuals in a social group that can be determinants of physical and psychological wellbeing [45,46]. Lower SES is a recognized risk factor for preterm birth in humans [47], and studies of African American women have found that experiences of racial discrimination also appear to be an independent risk factor [48]. Relative status might play a causal role in the chance of giving birth prematurely: a series of studies in the USA examining the consequences of changing SES on the risk of preterm birth showed that an intra-generational fall in SES was associated with a greater risk of preterm birth [49], with risk increasing with the degree of downward economic mobility, while a rise in SES was associated with a decrease in risk [50].

## 8. Preterm birth and reproductive life histories

We discuss above how spontaneous preterm birth occurs in response to a wide variety of triggers, and could be part of stereotyped response to adverse life conditions. Adverse early life conditions have also been associated in some females with an acceleration in the timing of puberty and an earlier age at menarche (age at first menstrual period). Setting this in a life-history context, it has been argued that across species there is commonly an association between earlier age at sexual maturation and reduced adult body size, and that this earlier age at maturation is associated with conditions during which there is a high risk of predation or death [18,51]. Studies of females who are born small or exposed to intra-uterine or infant stress show an earlier age at menarche [52], with severe family stress having a similar effect [53]. Based on these findings, Gluckman *et al.* argue that ‘based on the principles of developmental plasticity it becomes an appropriate response to accelerate the tempo of maturation in expectation of a shorter life expectancy’ [18, p. 120].

We conducted a systematic review to ask the question: does length of gestation affect the timing of puberty? [54]. We identified 16 studies of variable quality, of which 14 measured age at menarche. Of these, eight reported earlier menarche in preterm females, five found no difference and one showed later menarche in those born preterm. The largest study, involving a total of 2748 preterm females and 73 972 term controls, showed that those born preterm achieved menarche a median of 0.07 years earlier than those born at term [55], a finding in keeping with those of James *et al.*'s review [54] as a whole. A meta-analysis of studies that included a mean and standard deviation showed a mean age at menarche of 12.5 years for those preterm, and 12.6 years for those born at term, with overlapping 95% confidence intervals (electronic supplementary material, figure S1), suggesting that if there is a true biological effect of prematurity on age at menarche it is likely to be subtle.

## 9. Preterm birth and long-term phenotypic changes

Preterm birth can be associated with a range of cardiovascular, metabolic and psychological/behavioural modifications that extend into adulthood, even for individuals born at gestations close to term. One systematic review found that preterm birth was associated with a significantly higher systolic and diastolic blood pressure [56], potentially owing to factors such as reduced total nephron number [57]. However, the evidence for other markers of cardiovascular health is less consistent: some studies showed changes in intima-media thickness (an early sign of atheroma formation, and a risk for later atherosclerosis) [56] in preterm infants, while others showed no difference in features such as arterial stiffness [58], another risk factor for cardiovascular disease. Evidence for an association between preterm birth and long-term changes in glucose and insulin metabolism is again inconsistent. One large review [58] reported an association in early childhood between reduced insulin sensitivity (a risk factor for Type 2 diabetes) and preterm birth, but a reduction of the strength of this association in later childhood and adulthood. For adiposity, a meta-analysis identified a significant increase in low-density lipoprotein in infants born preterm [56], but no significant difference in adult BMI. For these cardiometabolic complications, there appears to be a strong modulating effect of infant nutrition, with preterm individuals who gain weight rapidly shortly after birth being more likely to develop features linked to an increased risk of the metabolic syndrome in later life [59].

In terms of mechanisms, studies suggest that induced changes in ‘epigenetic’ modifications including DNA methylation and histone modifications might be one mechanism by which adverse early life circumstances translate into phenotypic changes in adulthood. For example, altered DNA methylation has been reported in preterm infants at loci related to post-natal growth such as insulin-like growth factor 2 [60–63]. Another pathway by which preterm birth may exert long-term phenotypic change is through long-term effects on the HPA axis, for which altered activity is associated with a number of cardiovascular risk factors in adulthood [64]. Indeed, preterm infants have altered plasma cortisol levels in the first 2 years of life [65]. However, all studies examining cortisol metabolism in preterm infants are limited by the major confounding factor of maternal antenatal glucocorticoid administration (given to improve neonatal outcomes at a wide range of gestations) [64]. Thus, it is difficult to disentangle any effects of prematurity on the HPA axis from those resulting from exposure to large doses of synthetic glucocorticoids in the perinatal period.

A final way in which preterm birth might influence the adult phenotype is through psychological/behavioural changes—the hypothesis of the predictive adaptive response being that early exposure to an adverse environment leads to longer-term behavioural changes as an adaptation to predicted ongoing adverse conditions. In animal models, prenatal factors such as stress or toxicological exposures have been associated with the development of specific behavioural phenotypes [66]. Preterm birth is associated with a variety of neurodevelopmental disorders such as autism spectrum disorder, schizophrenia and anxiety/emotional disorders [4]. In addition to these severe sequelae, there is evidence for those born at a range of gestations of a ‘preterm behavioural phenotype’ [67], characterized by inattention, anxiety and social difficulty. The findings of a spectrum of milder changes in association with preterm birth are consistent with the idea that adversity in infancy or childhood may lead to stereotyped psychological/behavioural responses. For example, a large cohort study looking at childhood adversity (CA) and the risk of attention deficit hyperactivity disorder (ADHD) showed a dose–response relationship between the number of CA events and the risk of being diagnosed with ADHD [68].

## 10. Limitations of the evidence

What do the inconsistent findings outlined above imply for the theory of preterm birth forming part of an adaptive response to adverse life circumstances? An important consideration is that the evidence with which we are examining the hypotheses is inadequate. The assessment of gestational age is fraught with difficulty [69]: the studies referenced in this review will have used different methods to determine this exposure (birthweight, self-recalled date of LMP or ultrasound assessment). In addition to the information bias inherent in the assessment of gestation, the clinical syndrome of preterm birth is heterogeneous. While the spontaneous onset of labour is the most common single cause of preterm birth [39], factors precipitating early labour (such as infection or poor intra-uterine growth) may themselves be detrimental to the chances of immediate survival and later normal development and thus act as confounders. Equally, medical developments have led to both the delivery of infants at very early gestations, and survival at these gestations, so that studies looking at the consequences of preterm birth include a large proportion of individuals who in the past would not have survived infancy. Searching for evidence of an evolutionary process in a population with a heterogeneously defined exposure and a large number of confounders, even if preterm birth were to form part of a predictive adaptive response, might inevitably lead to conflicting and inconsistent results.

## 11. Can prematurity be seen as a PAR, or are other explanations more persuasive?

Drawing together the evidence, it appears that spontaneous preterm birth occurs commonly in humans, and can be triggered by a range of environmental factors. There are a number of associated longer-term phenotypic changes: not a marked temporal shift in the reproductive life-history trajectory, but potentially a re-programming of the cardiovascular and endocrine system, and effects on neurodevelopment. These, in the context of rapid weight gain, an obesogenic diet or other adverse life circumstances may lead to detrimental consequences for individuals.

So should preterm birth be seen mainly as a disease process, rather than one with any evolutionary consequences? The high global prevalence of preterm birth and the relatively high survival rates at gestations of greater than 32 weeks, even in low-income settings, would argue against this. However, the rapidly increasing mortality rates with decreasing gestation would suggest that the idea of preterm birth as an adaptation to an adverse environment is probably best examined in those born at moderately preterm gestations.

It is therefore possible that preterm delivery serves distinct purposes at different stages of gestation. What are now considered (thanks to the advent of modern technology) to be gestations at borderline viability and extremely preterm birth may be an outcome that prioritizes maternal survival, and primarily adaptive for the mother. An adaptive response for the fetus might be more relevant at later gestations, at which infant survival is more likely, and early delivery with adaptation to an adverse environment is the best possible outcome. Here prematurity could be seen as adaptive, but mainly in the immediate sense. Faced with adverse circumstances, preterm birth might form part of a strategy to enhance the chance of survival, but the consequent lack of investment in skeletal muscle, nephron number, body size and neuronal tissue is the inevitable, and detrimental, cost of responding to these conditions while ensuring survival. The long-term consequences of these immediate compromises may well be deleterious, but as outlined earlier, preterm birth offers a greater chance of survival than continuing a pregnancy in a compromised *in utero* environment. This view is consistent with Hales and Barker's proposal of an adaptive trade-off with relation to birthweight [70]: LBW or premature delivery is associated with higher rate of survival in adverse early life circumstances, but the consequent reduction of investment in, for example, nephron number [71] or capillary density [72] may later on in life lead to increased predisposition to cardiovascular disease.

Ultimately, validation of the predictive adaptive response as a plausible explanation for the phenotypic changes seen in response to adverse early life conditions will depend on the demonstration of positive effects on reproductive fitness accruing from an alteration in developmental trajectories, as seen in examples of the PAR in *Daphnia* or voles. Pointers towards this may come from clinical studies looking at the outcomes of early nutrition for preterm infants. It is known that excessive catch-up growth in this group (i.e. a mismatch between predicted and actual post-natal environments) can have detrimental consequences for the cardiometabolic health of these infants in later life [73], suggesting better outcomes for those who are appropriately matched to later environments. However, as we have highlighted throughout this review, high-quality datasets correlating gestation at birth with high granularity life trajectories, in the presence and absence of nutritional and other stresses, are currently lacking, limiting our ability to judge whether there may be long-term positive effects as a consequence of any early life programming.

## 12. Conclusion and public health implications

Preterm birth is a common, and potentially highly deleterious, complication of many pregnancies. Risk factors associated with preterm delivery correlate with adverse life conditions for mothers, which can take the form of nutritional, social or psychological disadvantage. In common with LBW, preterm birth appears to be sometimes, but not always, associated with a suite of phenotypic changes that may represent maladaptation to *unexpected* environmental conditions, or may instead be simply maladaptive in any context. As our understanding of the aetiology of spontaneous preterm birth is incomplete, and our ability to respond to the often devastating post-natal consequences of prematurity remains limited, focusing on the known preventable causes of preterm birth appears the most tractable approach at present to this substantial public health problem.

## Supplementary Material

Supplementary Figure 1.

## Data accessibility

This article has no additional data.
