# Supplementary material for: Preterm birth in evolutionary context: a predictive adaptive response?
Source: Philos Trans R Soc Lond B Biol Sci. 2019 Feb 25;374(1770):20180121. doi: 10.1098/rstb.2018.0121 (PMC6460087; doi:10.1098/rstb.2018.0121)
Supplement: Supplementary Figure 1. [file rstb20180121supp1.docx]

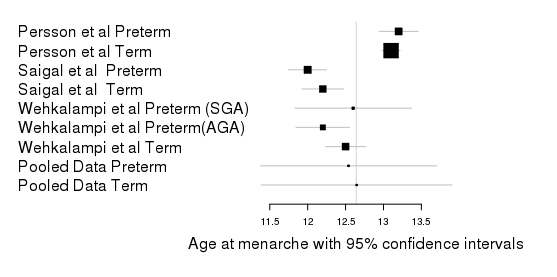


**Supplementary Figure 1**. Fixed effects meta-analysis of studies examining the timing of menarche after preterm birth, compared to controls born at term. Studies were taken from a systematic review examining the effect of preterm birth on the timing of puberty [1]. Only studies providing a mean and standard deviation were included [2–4].

**References**

1. James E, Wood CL, Nair H, Williams TC. 2018 Preterm birth and the timing of puberty: a systematic review. *BMC Pediatr.* **18**, 3. (doi:10.1186/s12887-017-0976-8)

2. Persson I, Ahlsson F, Ewald U, Tuvemo T, Qingyuan M, von Rosen D, Proos L. 1999 Influence of perinatal factors on the onset of puberty in boys and girls: implications for interpretation of link with risk of long term diseases. *Am. J. Epidemiol.* **150**, 747–55.

3. Saigal S, Stoskopf BL, Streiner DL, Burrows E. 2001 Physical growth and current health status of infants who were of extremely low birth weight and controls at adolescence. *Pediatrics* **108**, 407–15.

4. Wehkalampi K, Hovi P, Dunkel L, Strang-Karlsson S, Järvenpää A-L, Eriksson JG, Andersson S, Kajantie E. 2011 Advanced pubertal growth spurt in subjects born preterm: the Helsinki study of very low birth weight adults. *J. Clin. Endocrinol. Metab.* **96**, 525–33. (doi:10.1210/jc.2010-1523)
